# Genomics-Integrated Breeding for Carotenoids and Folates in Staple Cereal Grains to Reduce Malnutrition

**DOI:** 10.3389/fgene.2020.00414

**Published:** 2020-05-29

**Authors:** Kaliyaperumal Ashokkumar, Mahalingam Govindaraj, Adhimoolam Karthikeyan, V. G. Shobhana, Thomas D. Warkentin

**Affiliations:** ^1^Crop Improvement, Cardamom Research Station, Agricultural University, Pampadumpara, India; ^2^Crop Improvement program, International Crops Research Institute for the Semi-Arid Tropics, Hyderabad, India; ^3^Subtropical Horticulture Research Institute, Jeju National University, Jeju, South Korea; ^4^Department of Plant Sciences, College of Agriculture and Bioresources, University of Saskatchewan, Saskatoon, SK, Canada

**Keywords:** biofortification, nutri-genomics, cereal, folate, provitamin A, lutein, zeaxanthin, human nutrition

## Abstract

Globally, two billion people suffer from micronutrient deficiencies. Cereal grains provide more than 50% of the daily requirement of calories in human diets, but they often fail to provide adequate essential minerals and vitamins. Cereal crop production in developing countries achieved remarkable yield gains through the efforts of the Green Revolution (117% in rice, 30% in wheat, 530% in maize, and 188% in pearl millet). However, modern varieties are often deficient in essential micronutrients compared to traditional varieties and land races. Breeding for nutritional quality in staple cereals is a challenging task; however, biofortification initiatives combined with genomic tools increase the feasibility. Current biofortification breeding activities include improving rice (for zinc), wheat (for zinc), maize (for provitamin A), and pearl millet (for iron and zinc). Biofortification is a sustainable approach to enrich staple cereals with provitamin A, carotenoids, and folates. Significant genetic variation has been found for provitamin A (96–850 μg and 12–1780 μg in 100 g in wheat and maize, respectively), carotenoids (558–6730 μg in maize), and folates in rice (11–51 μg) and wheat (32.3–89.1 μg) in 100 g. This indicates the prospects for biofortification breeding. Several QTLs associated with carotenoids and folates have been identified in major cereals, and the most promising of these are presented here. Breeding for essential nutrition should be a core objective of next-generation crop breeding. This review synthesizes the available literature on folates, provitamin A, and carotenoids in rice, wheat, maize, and pearl millet, including genetic variation, trait discovery, QTL identification, gene introgressions, and the strategy of genomics-assisted biofortification for these traits. Recent evidence shows that genomics-assisted breeding for grain nutrition in rice, wheat, maize, and pearl millet crops have good potential to aid in the alleviation of micronutrient malnutrition in many developing countries.

## Introduction

Micronutrient and vitamin-deficiency-induced malnutrition is widely prevalent in South Asia and sub-Saharan Africa, affecting approximately two billion people worldwide. In the human diet, more than 50% of total calories come from major cereals, including rice, wheat, and maize, in developing countries and more than 70% in Southeast Asia and Africa. The green revolution contributed to remarkable increases in grain yield in these crops, which helped to prevent starvation in developing countries (Bouis and Welch, 2010). It is well known that cereal grains supply enough calories; however, these grains are inherently low in essential micronutrients, including carotenoids and folates (Bouis and Welch, 2010). The global production of rice is 769.4 m tons (from 167.2 m ha), wheat is 771.7 m tons (from 218.5 m ha), maize is 1134.7 m tons (from 197.2 m ha), and millet is 28.4 m tons (from 31.2 m ha) (Food and Agriculture Organization [FAO] et al., 2017), i.e., these crops play a critical role in food systems. Therefore, enhancing the nutritional quality of staple cereal crops is important for human health, particularly for resource-poor people in developing countries. Globally, 792.5 million people are malnourished, of which 780 million people live in developing countries (McGuire, 2015). Globally, two billion people suffer from hidden hunger due to inadequacies of micronutrients in their daily diet (Muthayya et al., 2013). Although major attention has been given to iron and zinc, in this review we also report on breeding efforts to improve concentrations of provitamin A, folate, and carotenoids.

Carotenoids are the second largest group of naturally occurring lipophilic pigments, following flavonoids, and at least 50 of them occur in plants. The most important carotenoids in food crops are β-carotene, α-carotene, β-cryptoxanthin, lutein, zeaxanthin, and lycopene. These carotenes are metabolized and converted to provitamin A (Davey et al., 2009). Humans are incapable of carotenoid biosynthesis, and we therefore depend on dietary carotenoid sources from plant-based foods (Fraser and Bramley, 2004). More than three million children in developing countries are affected by xerophthalmia, and 250,000–500,000 people become blind each year because of vitamin A deficiency (Food and Agriculture Organization [FAO] et al., 2017). The Recommended Dietary Allowance (RDA) of vitamin A for men and women is 900 and 700 μg Retinol Activity Equivalents (RAE)/day, respectively. For dietary provitamin A carotenoids, β-carotene, α-carotene, and β-cryptoxanthin RAEs have been set at 12, 24, and 24 μg, respectively (Institute of Medicine Food and Nutrition Board, 1998).

Folates act as cofactors in several metabolic functions, including the biosynthesis of nucleic acids and methylation of hormones, lipids, and proteins (Forges et al., 2007). Among many naturally occurring folates, cereal and pulse grains largely contain tetrahydrofolic acid (THF), 5-methyl-THF (5-MTHF), 10-formyl-THF (10-FTHF), and pteroylpolyglutamates (Jha et al., 2015; Ashokkumar et al., 2018b). Folate deficiency is a major problem for people from developing countries and can cause severe health issues, including impaired cognitive function, neural tube defects, and cardiovascular diseases (Ramos et al., 2005; McCully, 2007) as well as low birth weight, preterm delivery, and fetal growth retardation (Scholl and Johnson, 2000). Over 300,000 birth defects occur each year worldwide due to folate-deficiency-induced neural tube defects (Flores et al., 2014). Consumption of a folate-rich diet, fortification of foods with folic acid, and folic acid supplements can increase folate concentration in humans (Hefni et al., 2010). The RDA of folates is 400 μg for adults, 500 μg for lactating women, and 600 μg for pregnant women (Institute of Medicine Food and Nutrition Board, 1998).

Biofortification of staple crops through plant breeding and genomics integrated approaches is an effective strategy for delivering vitamins and nutrients to reduce micronutrient deficiencies in developing countries (Bouis, 2002; Welch and Graham, 2005). As urban development increasingly occupies fertile lands, the achievable agricultural production will be pushed toward marginal lands in developing countries. Enhancement of the nutritional value of staple crops through biofortification breeding might have a substantial impact on with their increased consumption worldwide. Increasing the availability of biofortified crops is a relatively straightforward approach to reach low-income people with limited access to healthy diets. Biofortification is a long-term, cost-effective, and sustainable approach to fight malnutrition in developing countries (Meenakshi et al., 2010). In the upcoming decades, the human population will increase in developing countries, and, with the altering climate conditions, food security will pose an increasing challenge (Das et al., 2013; Smith and Myers, 2018). Currently, the most common targeted micronutrients through biofortification breeding are iron, zinc, and carotenoids since these micronutrient deficiencies are common in children under the age of five and in pregnant and lactating women (Bouis and Welch, 2010). The World Health Organization (WHO) and Consultative Group on International Agricultural Research (CGIAR) aim to develop biofortified crops with enhanced nutrition (Bouis, 2000). To date, 36 biofortified varieties have been developed in maize, and these have reached 126,000 households in Zambia (Saltzman et al., 2017). The hybrid Pusa Vivek QPM nine Improved is the first biofortified maize variety in India with enhanced provitamin A. It was released in 2017 and is suitable for cultivation in nearly all states of India. Developing countries have included biofortification in their national agricultural nutrition strategies. For instance, India is the first country to prioritize biofortification and has set minimum standards for the release of pearl millet cultivars of 420 and 320 μg/100 g for iron and zinc, respectively. In this review, first major food sources and traits associated with carotenoids and folates have been discussed. In the next section, genetic variation and breeding strategies for enhancing the carotenoids and folates in major cereals (i.e., rice, wheat, maize, and pearl millet) have been summarized and discussed. In the final section we have discussed genomics integrated breeding and biofortification for carotenoids and folates as well as research gaps and future research directions.

## Important Food Sources of Carotenoids and Folates

**FIGURE 1:**
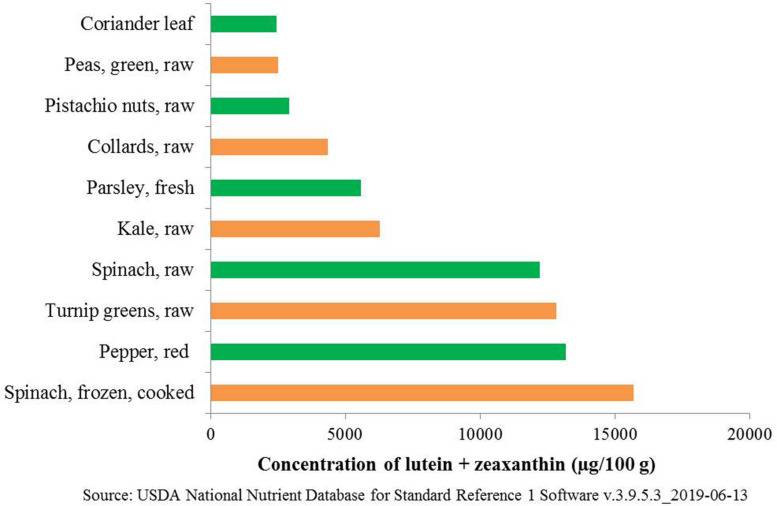
Top 10 food sources providing macular carotenoids (lutein + zeaxanthin).

Folate is also referred to as vitamin B_9_ and is involved in DNA and RNA synthesis. It is required to produce healthy red blood cells and is critical during periods of rapid growth, such as during pregnancy and fetal development. Carotenoids are essential for protecting eyes and bones and protecting against various types of cancer. Regular consumption of naturally available food sources can give a substantial quantity of folates, β-carotene, and macular carotenoids (lutein and zeaxanthin). However, the availability and affordability of such food sources are not possible in rural, poor, and remote areas in developing countries. The top ten food sources that are rich in (per 100 g) folates and carotenoids from earlier published reports and international food databases are summarized (Tables 1, 2 and Figure 1). Table 1 summarizes the percentage of recommended dietary allowance (% RDA) of folate, which is calculated based on a 100 g serving of each crop type expressed for adults, pregnant women, and lactating women. β-carotene is the precursor of provitamin A, and it is predominantly accumulated in fruits and vegetables (Ashokkumar et al., 2018a). Ten major food crops with the highest concentration of β-carotene are presented in Table 2. Among them, kale or leafy cabbage, sweet potato, and carrot have the greatest concentration of β-carotene. Continuous availability and accessibility of these sources at affordable prices is challenging; improving the nutritional value of locally produced and available foods is an appropriate way to address this issue.

**TABLE 1:** Folate-rich food sources available worldwide.

Sl. No.	Food source	Concentration (μg/100 g)	% RDA^§^			References
			Adult	Pregnant	Lactating women	
1.	Mung bean, raw	626.0	156.5	104.3	125.2	USDA–ARS (2012)
2.	Chickpea, raw	470.7	117.7	78.5	94.1	Jha et al. (2015)
3.	Common bean, raw	191.7	47.9	32.0	38.3	Jha et al. (2015)
4.	Lentil, green, raw	156.5	39.1	26.1	31.3	Jha et al. (2015)
5.	Soybean, green, raw	165.0	41.3	27.5	33.0	USDA–ARS (2012)
6.	Spinach, cooked	146.0	36.5	24.3	29.2	USDA–ARS (2012)
7.	Broccoli, cooked	108.0	27.0	18.0	21.6	USDA–ARS (2012)
8.	Bread wheat, raw	85.0	21.3	14.2	17.0	USDA–ARS (2012)
9.	Rice, pigmented, raw	51.0	12.8	8.5	10.2	Ashokkumar et al. (2018b)
10.	Corn, sweet, white, raw	46.0	11.5	7.7	9.2	USDA–ARS (2012)

*^§^The percentage of recommended dietary allowance (RDA) of folate concentration was calculated based on the serving of 100 g of each species. The United States (U.S.), Food and Nutrition Board, RDAs required 400 μg/day, 600 μg/day, and 500 μg/day for adult, pregnant and lactating women, respectively.*

**TABLE 2:** Rich food sources of provitamin A (μg/100 g)^a^.

Sl. No.	Food source	β-carotene (μg/100 g)	RAE (μg/day)^‡^	% RDA^§^			
				Children (1–3 years)	Children (4–8 years)	Men (>19 years)	Women (>19 years)
1.	Kale or leaf cabbage, raw	9226	768.8	256.3	192.2	85.4	109.8
2.	Sweet potato, raw	9180	765.0	255.0	191.3	85.0	109.3
3.	Carrot, raw	8836	736.3	245.4	184.1	81.8	105.2
4.	Squash, winter, butternut, raw	4226	352.2	117.4	88.0	39.1	50.3
5.	Collards, raw	3323	276.9	92.3	69.2	30.8	39.6
6.	Pepper, sweet, red, raw	2379	198.3	66.1	49.6	22.0	28.3
7.	Melon, cantaloupe, raw	1595	132.9	44.3	33.2	14.8	19.0
8.	Lettuce, romaine, raw	1272	106.0	35.3	26.5	11.8	15.1
9.	Apricots	664	55.3	18.4	13.8	6.1	7.9
10.	Peas, green, raw	432	36.0	12.0	9.0	4.0	5.1

*^a^Source: USDA–NCC Carotenoid Database for the US Foods-1998 published by Holden et al., 1999. ^§^Recommended dietary allowance (RDA) for vitamin A was calculated based on daily value (DV) of retinol activity equivalents (RAE) μg/day from 100 g serving of each species. The United States (U.S.), RDAs required RAE 300 μg/day and 400 μg/day for children aged 1–3 years and 4–8 years, respectively; 900 μg/day and 700 μg/day for adult men and women, respectively. ^‡^RAE was calculated by 12 μg dietary β-carotene converted to 1 μg retinol (REA ratio 12:1).*

## Traits Associated With Carotenoids and Folates

The growing food markets pay close attention to grain nutritional quality due to the mounting health concerns among consumers. Yellow to orange pigmented grain types are positively correlated with carotenoid concentration in maize (da Silva Messias et al., 2014). Carotenoids are located in amyloplasts in maize. Lutein is the major carotenoid present in the grains of wheat (Ramachandran et al., 2010), pulses (Ashokkumar et al., 2014, 2015), oilseeds (McGraw et al., 2001), and spices (Ashokkumar et al., 2020). The seeds of wild-type maize chiefly accumulate lutein, followed by zeaxanthin, xanthophyll, and trace amounts of β-carotene (Janick-Buckner et al., 1999). Lutein and zeaxanthin are the major carotenoids in millets, with lutein being the predominant in white millet, while zeaxanthin is the main carotenoid in red millet (McGraw et al., 2001). Similarly, the yellow kernel color of maize was positively correlated with non-provitamin carotenoids lutein and zeaxanthin (Muthusamy et al., 2015). A red-pigmented rice grain variety accumulated two-fold higher folate concentration than that found in white rice grains (Ashokkumar et al., 2018b). Abscisic acid (ABA) accumulation in grains is one of the important traits associated with carotenoid concentration (Maluf et al., 1997). Sometimes, reducing the antinutrient factors, such as phytic acid, may enhance the nutritional quality and bioavailability of cereals (Bohn et al., 2008; Tamanna et al., 2013). This approach has been effectively used to enhance the nutrition of maize grown for animal feed (Raboy, 1996). The highest accumulation of total carotenoids in wheat grain was reported at 12–15 days after anthesis and thereafter the level of accumulation declined (Graham and Rosser, 2000).

## Genetics and Genetic Variation of Carotenoids and Folates in Cereals

Genetic analysis of carotenoids offers expedient directions to breeders initiating further breeding events. However, limited information is available on the genetic control of carotenoid concentration in staple cereal crops (Table 3). Yellow pigment concentration (YPC) in wheat and β-carotene, α-carotene, β-cryptoxanthin, and provitamin A in maize endosperms are largely controlled by additive genetic variance (Elouafi et al., 2001; Halilu et al., 2016). These complex traits may be linked to genotype-dependent and environmental factors. Grain yield and carotenoid concentration were predominantly controlled by non-additive gene actions in maize (Halilu et al., 2016). Furthermore, earlier investigations reported that carotenoids and its related compounds were controlled by both additive and non-additive gene action in maize endosperm (Chander et al., 2008). Babu et al. (2013) noticed that partial dominant and partial recessive gene action was in play in maize for the genes *LCYE-50TE* and *crtrB1-30TE*, respectively. The superiority of additive gene action and non-additive gene action suggested the application of recurrent selection and heterosis breeding followed genetic improvement of a particular trait in cereal crops.

**TABLE 3:** Genetic control of carotenoid concentration in major cereal grains.

Crop	Trait	Gene effects	References
Wheat	Yellow pigment concentration	Additive	Clarke et al. (2006)
Wheat	β-carotene	Digenic epistasis (additive × dominance)	Santra et al. (2005)
Maize	β-carotene	Incompletely dominant	Hauge and Trost (1928)
Maize	Carotenoids	Additive	Kandianis et al. (2013)
Maize	β-carotene	Additive	Jittham et al. (2017)
Maize	Provitamin A	Non-additive	Halilu et al. (2016)
Pearl millet	β-carotene	Non-additive	Khangura et al., 1980
Sorghum	β-carotene	Additive	Fernandez et al. (2008)

Heritability estimates are mainly used for the determination of genotypic proportion of the trait, which favors the estimation of the effect of selection. If a particular trait has a higher heritability value, that trait might be modified by proper selection methods. Conversely, lower heritability values indicated that those selection methods are not suitable for that particular trait. However, various researchers remarked that low to high heritability values were observed for carotenoids in maize. The heritability of YPC ranged from low (11%) to high (69%) in wheat (Elouafi et al., 2001; Clarke et al., 2006). Broad-sense heritability (*H*^2^) was observed for lutein (61.49%), zeaxanthin (58.91%), and β-carotene (67.37%) in maize. Studies also noted narrow sense heritability (*h*^2^) for lutein (19.00%) and zeaxanthin (18.09%) (Halilu et al., 2016). However, higher broad sense heritability was detected for lutein and zeaxanthin (Chander et al., 2008), and medium heritability values were observed for provitamin A (Wong et al., 2004). Genetic studies for gene action and heritability estimates are essential before initiating biofortification breeding programs for provitamin A and folates since heritability and gene action could be varied for different plant materials and environmental factors. Additionally, the investigation of gene action is imperative to design breeding programs.

In order to breed varieties with enhanced carotenoid and folate concentrations, information on the magnitude of genetic variation for carotenoids and folate in rice, wheat, maize, and pearl millet is needed. The variability for carotenoid and folate concentrations that have been recorded in the available genetic resources is summarized in Table 4. Genetic variation for β-carotene ranged from 96–850 μg/100 g in wheat, and 0.0–1780 μg/100 g in maize (Santra et al., 2005; Badakhshan et al., 2013; Muthusamy et al., 2014, 2015). In a study of 100 maize inbred lines, lutein and zeaxanthin concentrations ranged in the order of 20–1130 μg/100 g and 20–2000 μg/100 g, respectively. The highest lutein (1130 μg/100 g) and zeaxanthin (2000 μg/100 g) contents were recorded in two maize genotypes, namely, HP180-25 and CML161. According to Ortiz-Monasterio et al. (2007), 5–30% total carotenoids were provitamin A carotenoids while, β-carotene and β-cryptoxanthin were around 21 and 27% of the total concentrations of kernel carotenoids of yellow maize genotypes, respectively (Suwarno et al., 2014). These studies show that substantial genetic variability is present in the maize genetic resources for provitamin A and non-provitamin A concentrations of carotenoids, which could be used for the development of biofortified maize varieties/hybrids.

**TABLE 4:** Range of carotenoid and folate concentrations in the available genetic resources of major cereal grains.

Crop	Genotypes evaluated	Nutrient trait	Concentration (μg/100 g)	References
Rice	4 genotypes	Folate	11.0–51.0	Ashokkumar et al. (2018b)
Wheat	130 winter wheat genotypes	Folate	36.4–77.4	Piironen et al. (2008)
Wheat	20 spring wheat genotypes	Folate	32.3–74.1	Piironen et al. (2008)
Wheat	10 durum wheat	Folate	63.7–89.1	Piironen et al. (2008)
Wheat	82 wheat accessions	β-carotene	96.0–169.0	Badakhshan et al. (2013)
Wheat	5 genotypes	β-carotene	300.0–850.0	Santra et al. (2005)
Maize	12 inbred lines	Provitamin A	738.0–1359.0	Zunjare et al. (2018)
Maize	111 inbred lines	Total carotenoid	650.0–6730.0	Sivaranjani et al. (2013)
Maize	105 inbred lines	Lutein	20.0–1130.0.0	Muthusamy et al. (2015)
Maize	105 inbred lines	Zeaxanthin	20.0–2000.0	Muthusamy et al. (2015)
Maize	105 inbred lines	β-carotene	0.0–1500.0	Muthusamy et al. (2015)
Maize	105 inbred lines	β-cryptoxanthin	10.0–330.0	Muthusamy et al. (2015)
Maize	27 inbred lines	β-carotene	130.0–1780.0	Muthusamy et al. (2014)
Maize	64 inbred lines	Total carotenoids	558.0–390.0	Safawo et al. (2010)
Maize	64 inbred lines	β-carotene	12.0–474.0	Safawo et al. (2010)
Pearl millet	10 F_5_ progeny lines	β-carotene	129.0–173.0	Jiji et al. (2017)
Pearl millet	10 F_5_ progeny lines	Total carotenoids	329.0–810.0	Jiji et al. (2017)
Sorghum	11 genotypes	β-carotene	56.0–113.0	Reddy et al. (2005)
Sorghum	121 RILs	Lutein	8.0–63.0	Fernandez et al. (2008)
Sorghum	121 RILs	Zeaxanthin	6.1–102.0	Fernandez et al. (2008)

Pearl millet has limited concentrations of β-carotene, but a few accessions were identified with higher levels. For instance, genotype PT 6129 was high in β-carotene (241.7 μg/100 g), and such a line is useful to breed carotenoid rich varieties (Aarthy et al., 2011). Additionally, genetic variability is being explored in sorghum through the yellow endosperm lines which are available in the germplasm collections of the International Crops Research Institute for the Semi-Arid Tropics (ICRISAT), Patancheru, Hyderabad, India. The β-carotene concentrations of sorghum lines ranged from 56–113 μg/100 g, six lines, namely, IS 7684, IS 7776, IS 24703, IS 24868, IS 24883, and IS 26886, having an average of 85 μg (Reddy et al., 2005).

In terms of carotenoids in wheat seeds, lutein, zeaxanthin, and β-cryptoxanthin were predominant in the germ, while the endosperm had predominantly lutein, followed by β-cryptoxanthin and zeaxanthin (Table 5). For instance, the lutein concentration of wheat endosperm and germ varied significantly from 15.5 to 70.7 μg and 43.1 to 193.7 μg in 100 g, respectively (Adom et al., 2005; Masisi et al., 2015). The total carotenoids in wheat ranged from 170.1 to 227 μg/100 g in endosperm and 945 to 1029 μg/100 g in bran or germ (Ndolo and Beta, 2013). Interestingly, maize endosperm had substantial concentrations of zeaxanthin (1367.1 μg/100 g) and total carotenoids (1417.1–3135.2 μg/100 g).

**TABLE 5:** Grain localization of carotenoids and folates in major cereals.

Crop	Genotypes evaluated	Nutrient trait	Concentration (μg/100 g)			References
			Endosperm	Germ	Aleurone	
Wheat	5 genotypes	Lutein	36.9–70.7	164.1–191.7	–	Adom et al. (2005)
Wheat	5 genotypes	Zeaxanthin	1.6–2.7	19.4–26.2	–	Adom et al. (2005)
Wheat	5 genotypes	β-cryptoxanthin	3.5–4.4	8.91–10.0	–	Adom et al. (2005)
Wheat	1 genotype	Lutein	15.5	43.1	2.2	Masisi et al. (2015)
Wheat	1 genotype	Zeaxanthin	0.7	21.5	21.2	Masisi et al. (2015)
Wheat	4 genotypes	Total carotenoids	171.0.–227.1	845.1–987.1	–	Ndolo and Beta (2013)
Maize	1 genotype	Lutein	136.9	7.2	16.1	Masisi et al. (2015)
Maize	1 genotype	Zeaxanthin	1367.1	98.9	35.8	Masisi et al. (2015)
Maize	4 genotypes	Total carotenoids	1417.1–3135.2	33.3–53.6	–	Ndolo and Beta (2013)

Few studies have been conducted for the evaluation and identification of plant genetic resources for folate enhancement in cereal grains. This is likely due to the complexity, stability, and cost of folate concentration assays. Folate concentration was double in red-pigmented rice (Nootripathu) compared to non-pigmented rice genotypes (IR 20, N 22, and Pusa Basmati-1), and it ranged from 11 to 51 μg/100 g (Ashokkumar et al., 2018b). Piironen et al. (2008), assessed the total folate concentration in 160 genotypes of winter, spring, and durum wheat, and it ranged from 32.3 to 89.1 μg/100 g, with the greatest range evident in durum (63.7–89.1 μg/100 g). Their growing environments significantly influenced total folate concentration of winter wheat genotypes, more so than the genetic factors (Kariluoto et al., 2010). Variation for folates in maize, sorghum and pearl millet was not reported among the available genetic resources. Hence, further studies are needed to investigate the folate concentrations in grains of those major cereals. The rich sources of germplasm and their use for the genetic improvement and grain localization of carotenoids and in major cereal grains are described (Table 6).

**TABLE 6:** Available genetic resources for carotenoids and folate improvement in major cereal grains.

Crop	Genotype	Nutrient trait	Concentration (μg/100 g)	RAE (μg/day)^‡^	% RDA^§,†^				References
					Children (1–3 years)	Children (4–8 years)	Men (>19 years)	Women (>19 years)	
Rice	Nootripathu	Folates	51.0		34.0	25.5	12.8	12.8	Ashokkumar et al. (2018b)
Maize	HP704-22	Provitamin A	1605.0	133.8	44.6	33.4	14.9	19.1	Zunjare et al. (2018)
Maize	HP704-23	Provitamin A	1528.0	127.3	42.4	31.8	14.1	18.2	Zunjare et al. (2018)
Maize	HP465-41	Provitamin A	1550.0	129.2	43.1	32.3	14.4	18.5	Muthusamy et al. (2015)
Maize	HP465-30	Provitamin A	1510.0	125.8	41.9	31.5	14.0	18.0	Muthusamy et al. (2015)
Maize	HP180-25	Lutein	1130.0	–		–		–	Muthusamy et al. (2015)
Maize	CML161	Zeaxanthin	2000.0	–		–		–	Muthusamy et al. (2015)
Maize	HPLET-03-36	Total carotenoid	6730.0	–		–		–	Sivaranjani et al. (2013)
Maize	HPLET-03-37	Total carotenoid	6320.0	–		–		–	Sivaranjani et al. (2013)
Maize	HPLET-03-35	Total carotenoid	5990.0	–		–		–	Sivaranjani et al. (2013)
Maize	BLSB-RIL17	Total carotenoid	5700.0	–		–		–	Sivaranjani et al. (2013)
Maize	BLSB-RIL43	Total carotenoid	5670.0	–		–		–	Sivaranjani et al. (2013)
Maize	HPLET-03-41	Total carotenoid	5610.0	–		–		–	Sivaranjani et al. (2013)
Maize	BLSB-RIL95	Total carotenoid	5090.0	–		–		–	Sivaranjani et al. (2013)
Maize	UMI176	β-carotene	580.0	48.3	16.1	12.1	5.4	6.9	Safawo et al. (2010)
Pearl millet	PT 6129	β-carotene	241.7	20.1	6.7	5.0	2.2	2.9	Aarthy et al. (2011)
Pearl millet	PT 6129	Total carotenoid	899.0	–		–		–	Jiji et al. (2017)
Sorghum	PI 585351	Total carotenoid	234.3	–		–		–	Shen et al. (2017)

*^‡^RAE was calculated by converting 12 μg of dietary β-carotene into 1 μg of retinol (REA ratio 12:1); ^§^Recommended dietary allowance (RDA) for vitamin A was calculated based on daily value (DV) of retinol activity equivalents (RAE) μg/day from 100 g serving of each species. The United States (U.S.), RDAs required RAE 300 μg/day and 400 μg/day for children aged 1–3 years and 4–8 years, respectively; 900 μg/day and 700 μg/day for adults of both men and women, respectively.*

## Breeding for Increased Carotenoid and Folate Concentration

**FIGURE 2:**
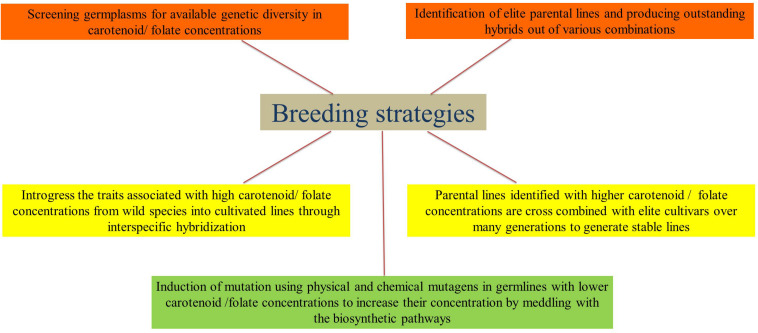
Breeding strategies for enhancing carotenoids and folates in cereals.

The breeding strategies that are widely used to improve the carotenoid and folate concentrations in cereals are presented in Figure 2. Rice does not contain adequate amounts of carotenoids (i.e., β-carotene), which the human body could convert into vitamin A. Conventional breeding strategies have not been successful in increasing the β-carotene contents in rice endosperm. This is due to the fact that there is no genotype/cultivar that can synthesize carotenoid in the endosperm of the seed and the available contents are very low. Tan et al. (2004) showed that brown rice contains carotene and/or lutein, but the polishing process considerably reduces its nutritional value. In this respect, genetic engineering offers opportunities to improve the levels of provitamin A in rice grain. The control of the expression of ferritin through its control on the glutelin promoter has been successful in increasing nutritional levels in the whole and polished grains of rice. Similar principles have been used in the development of golden rice (Datta et al., 2007; Paine et al., 2005; Ye et al., 2000). Currently, no rice genotype has been enhanced for β-carotene content through traditional breeding strategies. It is obvious that there is huge potential in the exploitation of genetic variability of the carotenoid content in rice grains. However, the bioavailability of β-carotene should be studied in greater depth. In the case of folates, very few attempts were made to characterize the folate profile in rice by screening the germplasm. Blancquaert et al. (2015) screened 12 rice cultivars and found a two-fold difference (up to 70 μg/100 g) in the total grain folate content. The natural range of folate concentrations was determined in 78 rice varieties and both in milled (up to 78 mg/100 g) and whole grains (up to 111 μg/100 g), the contents exhibited an eight-fold difference (Yu and Tian, 2018). In all diverse accessions of rice germplasms around the world, an even more extensive screening for folate would bring out higher levels of variation in folate contents. This could be utilized in breeding programs for enhancing the folate contents in rice.

Natural genetic variability is very low for β-carotene contents in wheat grains. Lutein is the most common carotenoid in tetraploid wheat grains, whereas hexaploid wheat grains contain minimal levels of total carotenoids (Abdel-Aal el et al., 2007; Lachman et al., 2013). The durum wheat variety HI 8627 with high provitamin A was released by IARI, India, in 2005. The “Yellow pigment” is primarily caused by lutein, which is one of the significant factors in the enhancement of quality traits. Both lutein and anthocyanins are antioxidants in nature, which provokes a lot of interest in the research community. Black grained wheat cultivars and colored wheat cultivars are already being exploited in many breeding programs around the globe and they are rich in protein and selenium (Li et al., 2006). The purple wheat cultivar Indigo, which was released in Austria in 2006 (Eticha et al., 2011), the purple wheat cultivar PS Karkulka of Slovakia in 2014, and purple, blue, and black white lines of India in 2017 (Garg et al., 2016) are major sources of carotenoids in wheat breeding. Poutanen et al. (2008) evaluated the genetic variation for folates in the Health Grain wheat diversity screen with whole and milled wheat grains. Around 150 varieties of hexaploid, diploid, and tetraploid wheat showed two-fold variation in folate content (up to 77 μg/100 g) in whole grains (Piironen et al., 2008; Ward et al., 2008). Environmental effects cause variations in folate contents indicating low heritability and high G × E interactions in diverse varieties (Shewry et al., 2010). Induced chemical or physical mutagens could be utilized to identify mutants with greater folate contents.

Most of the breeding programs targeted to improve the provitamin A in maize aims at developing high yielding, provitamin A-enriched maize cultivars that fetch profit for the farmers and also promise customer preference and may ensure the effective reduction of vitamin A deficiency (Bouis and Welch, 2010). The simultaneous improvement of provitamin A carotenoids and grain yield is easily attainable. This is due to the weak correlation between provitamin A and agronomic performance. Other factors, like the relatively high heritability of the trait, the mode of inheritance (additive genetic effects), and the genetic control of provitamin A, are also accountable (Suwarno et al., 2014; Menkir et al., 2018; Ortiz-Covarrubias et al., 2019). So far, the enhancement of provitamin A is mostly focused on the selection of β-carotene content. A target of 1500 μg/100 g of β carotene equivalents was set for breeders beyond which there occurs an increasingly marked effect on the human health (Hotz and McClafferty, 2007). Around 1,500 genotypes were screened for their carotenoid contents by various researchers, resulting in about 200–300 μg/100 g in their profiles (Ortiz-Monasterio et al., 2007). Among these germplasms, only a few lines of the temperate zones contained target level in their seeds (Menkir et al., 2008). In the meantime, the tropical and sub-tropical inbred lines possessed very low levels of provitamin A when compared with the breeding target in maize (Bouis et al., 2011). It demands the necessity and the initiation of searching for novel sources of favorable alleles to boost provitamin A concentration to new levels. Taleon et al. (2017) and Sowa et al. (2017) emphasized the application of breeding for provitamin A carotenoids that would increase β-cryptoxanthin rather than β-carotene, as β-carotene has lower stability, while β-cryptoxanthin ensures higher bioavailability and bioefficacy to β-carotene (Schmaelzle et al., 2014; Menkir et al., 2018). Breeding programs with this vision have already been initiated, resulting in inbreds that are being used in the improvement of new hybrids and synthetics. So far, most of the pearl millet breeding programs are targeted for improving grain iron and zinc concentration and yield related traits. Limited breeding efforts have been made thus far to explore the genetic variation of carotenoids and folates in pearl millet. Current circumstances demand carotenoid- and folate-rich donor lines for pearl millet breeding, and large numbers of germplasms must therefore be screened.

Typically, plant breeders use bi-parental populations for identification of QTL and development of varieties for the traits of interest. Many varieties developed of rice, wheat, maize, and pearl millet are based on single crosses between two parents. However, a higher number of parents and initial crosses will lead to a better dissection of complex traits. Thus, breeders recently introduced new experimental design namely multiparent populations, which provide significant benefits for genetic and QTL studies in plants. One of the most popular multiparent populations is the multiparent advanced generation intercross (MAGIC) population. The major goal of constructing MAGIC populations is to encourage intercrossing and shuffling of the genome into a single line (Huang et al., 2012; Holland, 2015). It is a diverse population with high recombination, thus providing excellent breeding materials to genetic and QTL mapping studies for complex traits such as carotenoids and folates. MAGIC populations have identified multiple loci and demonstrated the genetic complexity of the grain micronutrients (Fe and Zn), cooking quality, and agronomic traits (Holland, 2015; Descalsota et al., 2018; Ponce et al., 2018) in rice. Similarly, genetic properties of the MAGIC populations have also been detected in maize and wheat, and their benefits in detecting the complex traits have been confirmed by many researchers (Huang et al., 2012; Verbyla et al., 2014; Holland, 2015; Chen et al., 2016; Butrón et al., 2019). However, no study has been published that investigates carotenoids and folates using a MAGIC population design in cereals. ICRISAT has been developing a MAGIC population for various traits, including grain micronutrients (unpublished). Thus, it is a highly prioritized research area in which to work in the future for cereal-based national and international research organizations.

## Genomics-Enabled Breeding Approaches for Improving Carotenoids and Folates

**FIGURE 3:**
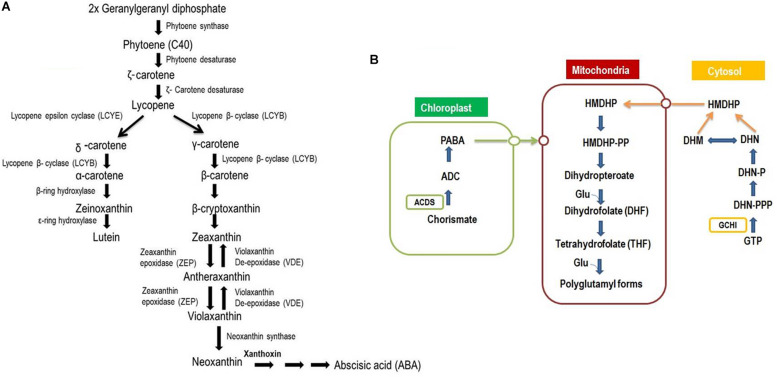
Biosynthesis of carotenoids and Folates in plants. **(A)** Carotenoids biosynthesis and subsequent influential of phytohormones and provitamins. Footnotes: The first committed step in carotenoid biosynthesis is the condensation of two molecules of Geranylgeranyl diphosphate (GGPP) by phytoene synthase (*PSY*) to form phytoene (C40). The colorless phytoene is subsequently desaturated to give zeta-carotene and lycopene. Desaturation of phytoene occurs by two enzymes, phytoene desaturase (*PDS*) and zeta-carotene desaturase (*ZDS*), which are required to form lycopene. A major branch point occurs after lycopene synthesis when cyclization mediated by the enzymes lycopene-b-cyclase (*LCYB*) and lycopene-3-cyclase (*LCYE*) gives rise to α-carotene and β-carotene. α-carotene is acted upon by a β-ring hydroxylase to form zeinoxanthin, which is then hydroxylated by a ε-ring hydroxylase to produce lutein. β-carotene can be hydroxylated β-carotene hydroxylase (*CRTRB*) in a two-step reaction to zeaxanthin, with β-cryptoxanthin as an intermediate product. Zeaxanthin can be epoxidized to violaxanthin, and a set of light- and dark-controlled reactions, known as the xanthophyll cycle, rapidly optimize the concentration of violaxanthin and zeaxanthin in the cell through the action of zeaxanthin epoxidase (*ZEP*) and violaxanthin de-epoxidase (*VDE*), respectively, via antheraxanthin. Violaxanthin undergoes synthesis by the enzyme neoxanthin synthase to form neoxanthin and as precursor of the plant hormone abscisic acid. **(B)** Biosynthetic pathway of folates (Adapted by DellaPenna, 2007). Footnotes: The pteridine pathway leading to hydroxymethyldihydropterin (HMDHP) is shown in blue, the pathway leading to p-aminobenzoate is shown in green, and steps localized in the mitochondria are in black. Open circles indicate possible transporters. Red arrows indicate the two enzymes GTP-cyclohydrolase I (*GCHI*) and aminodeoxychorismate synthase (*ADCS*). DHN, dihydroneopterin; -P, monophosphate; -PP, pyrophosphate; -PPP, triphosphate; DHM, dihydromonapterin.

Genomics research in cereals has substantially improved our knowledge of the QTLs/genes and biochemical pathways involved in carotenoids and folates in cereals (Figure 3). Second- and third-generation sequencing technologies have been game changers for genomics research and contributed to completion of the reference genome sequences for major cereal crops including rice (Yu et al., 2002), wheat (Brenchley et al., 2012), maize (Schnable et al., 2009), and pearl millet (Varshney et al., 2017). This genomic revolution has led to a pronounced increase in our knowledge of cereal genomics and our understanding of the structure and behavior of the cereal genomes. So far, an impressive number of genomic resources including detailed high-density genetic maps, cytogenetic stocks, contig-based physical maps, and deep coverage, and large-insert libraries have been developed in cereal crops (Muthamilarasan and Prasad, 2016). More interestingly, the genomic resources from a model or major cereal species (i.e., rice, maize, and wheat) also have potential that can be exploited for the development of minor cereals through comparative genomics approaches (Varshney et al., 2006). The transfer of genomic information and techniques from model or major to minor cereals provides detailed information about the genetic diversity of the crop and assists in the identification of the potentially beneficial variants in minor cereals. It also provides a greater chance for the identification of favorable alleles and the cloning and transfer of favorable alleles within the species.

Genomics offers tools to improve the contents of carotenoids and folates in cereals through advanced breeding techniques. Refining the breeding strategies through marker-assisted selection (MAS) is significantly improving the effectiveness of breeding for the enhancement of carotenoids and folates in cereals. The availability of the whole genome sequence data of major cereals enables the development of molecular markers. Among the different types of molecular markers, simple sequence repeats (SSRs) and single nucleotide polymorphisms (SNPs) markers are considered to be the markers of choice for a variety of applications, mainly in marker-assisted breeding (MAB). Besides, the genic or functional markers developed from the transcribed regions of the genome also act as ideal markers for MAB and as a major resource for assessing the functional variation in natural or breeding populations, in cereals. Expressed sequence tags (ESTs) or gene sequences have also been used to find SSRs/SNPs, and genic molecular markers have also been developed in cereals (McCouch et al., 2002). Fortunately, the information about SSR and SNP markers are available in the public domain for crop such as rice (McCouch et al., 2002), wheat (Jaiswal et al., 2017), maize (Sharopova et al., 2002) and pearl millet (Senthilvel et al., 2008). Thus, MAB in cereals has become standard procedure and many researchers to improve the levels of carotenoids and folates in cereals are pursuing these markers. Capitalizing on the genome-wide marker data, linkage-map-based QTL mapping, genome-wide association studies (GWAS), and genomic selection (GS) have become powerful tools to dissect the QTL and investigate trait-allele associations in cereals. To date, several QTLs/genes associated with carotenoids and folates in cereals were identified using linkage-map-based QTL mapping and GWAS. In particular, GWAS effectively pinpoints the genes that play a key role in the biosynthesis of carotenoids and their accumulation, and to find out the variation in the alleles at the concerned loci that are related to the biosynthesis of carotenoids in maize and wheat (Yan et al., 2010; Colasuonno et al., 2017). However, the nutritional traits like carotenoids and folates are quantitative and governed by minor QTLs that are responsible for the large phenotypic variation, including epistatic interactions. In this case, GS can capture both minor effects of QTL and epistatic interaction effects, so it could be a highly useful strategy in trait genetic gains of crop breeding programs. GS determines the genetic potential of an individual based upon the genomic estimated breeding values (GEBVs) instead of identifying the specific QTL (Robertsen et al., 2019). In the process of enhancing various complex traits, genomic selection has been used in cereals and other commercial crops. Still, the benefits of GS have not been utilized for the improvement of folates and carotenoids in cereals. This should provoke interest among the researchers working for upgrading the nutritional status of the major cereal crops in developing countries. So far, many QTLs/genes associated with carotenoids (provitamin A, lutein, and zeaxanthin) and folates have been identified in major cereals (i.e., rice, maize, and wheat). However, all of these QTLs/genes are not equally effective in the production of carotenoids and folates. Therefore, some of the important QTLs/genes that are identified so far are summarized and discussed here.

## QTLs and Candidate Genes for Carotenoids and Folates

### (a) Rice

QTL and genes have recently been identified for folate contents in rice through mapping studies, but in the case of carotenoids, no such information is available. In experiments with recombinant inbred lines and backcrossed lines of milled rice, several major QTLs were identified to be associated with a higher level of folate. Dong et al. (2013) identified three QTLs, *qQTF-3-1, qQTF-32*, and *qQTF-3-3*, located on chromosome 3, which contributed 7.8, 11.1–15.8, and 25.3% of the variation in folate concentration. Three genes are associated with these QTLs, i.e., a rice homologue of plastidial folate transferase of *Arabidopsis*, a rice homologue of human folate hydrolase, and the serine hydroxymethyl transferase gene. When these newly identified QTLs associated with high folate are used in the synthesis of commercial varieties with high folate concentrations, there will be a larger wealth of knowledge about folate and its metabolism, regulation, and accumulation in grains (Yu and Tian, 2018).

### (b) Wheat

Genetic analyses based on molecular markers have mapped major QTL for YPC on chromosome 7. Minor QTLs, associated with YPC, were detected on almost all chromosomes of the wheat genome. Some of these QTLs are stable, and they may be suitable for MAS in breeding programs. Two major QTLs were on chromosomes 3A and 7A, with 13 and 60% of the phenotypic variance, respectively (Parker et al., 1998). The QTL on chromosome 7A found to be closely related to an AFLP marker *Xwua26-7A.4* (Parker and Langridge, 2000), which was later transformed into an STS marker. Further, QTL that controls YP concentration of the kernels was detected on chromosome 7A with 12.9–37.6% of phenotypic variance in five different locations (Zhang et al., 2006). The YPC genes that encode phytoene synthase (*Psy*) have been mapped on the homologous groups of chromosomes 7 and 5 in wheat (Pozniak et al., 2007). There is an association between the loci of *Psy-B1*, which co-segregated with a QTL for endosperm color on 7B. Through *in silico* cloning, He et al. (2008) have categorized the association of the YPC in wheat grain across the full-length of the sequence of the genomic DNA sequence of a *Psy-A1*, which is linked to the SSR marker, *Xwmc809*, on the long arm of chromosome 7A with 20–28% of the phenotypic variance for the YP concentrations. Zhang et al. (2009) identified four QTLs namely, *QYpc-1A, QYpc-1B, QYpc-4A*, and *QYpc-7A*, for the YP concentration on chromosomes 1A, 1B, 4A, and 7A, which explained 1.5–33.9% of the phenotypic variance. Blanco et al. (2011) investigated the recombinant inbred line population arising from wheat cultivars Latino and Primadur, and they found that the QTLs linked with the concentration of YP and individual carotenoid compounds, namely, lutein, α-carotene zeaxanthin, β-cryptoxanthin, and β-carotene, were present on the same genomic regions of chromosomes 2A, 3B, 5A, and 7A. A single locus *called Lute*, controlling the lutein esterification on the short arm of chromosome 7D in wheat (Ahmad et al., 2015). The syntenic region of the rice genome contained a GDSL-like lipase gene. The sequences of wheat that are similar to this gene were mapped at the same locus of *Lute*. Folate variation in wheat accessions is very limited; almost no information is available on the folate QTLs and genes in wheat. Thus, researchers are trying to identify novel QTLs and markers that are closely associated with folate for marker-assisted breeding in wheat.

### (c) Maize

Several QTLs and genes related to carotenoids (provitamin A, lutein, and zeaxanthin) and folates have been reported in maize using different mapping approaches. *Yellow* 1 (*Y1*) gene encoding *PSY1* (phytoene synthase1) and is positioned on chromosome 6 in maize (Buckner et al., 1996). The gene *PSY1* was studied through association mapping in two different populations of maize. This gene has two alleles that are responsible for the differences in total carotenoids. Further, QTL mapping was carried out in one segregating population and lines that are polymorphic for genomic regions within *PSY1* were studied for expression analysis. Two functional sites that are concerned with the total carotenoid concentration of maize contributed 7 and 8% of the genetic variation (Fu et al., 2013).

Phytoene desaturase (*PDS*) and zeta-carotene desaturase are the enzymes that desaturate phytoene into lycopene. Lycopene is the first pigment that is produced in maize (Li et al., 1996). PDS is associated with viviparous 5 (*vp5*) that was mapped on chromosome 1. It was found that ζ−carotene isomerase (*Z−ISO*) was encoded by locus *y9* (Chen et al., 2010) and located on chromosome 7. Without the presence of Z−ISO, no provitamin A carotenoids could be synthesized in the endosperm (Matthews et al., 2003; Chen et al., 2010). Furthermore, 30 QTLs for carotenoid composition were also identified (Wong et al., 2004; Chander et al., 2008). A few of these are tightly linked to the biosynthetic pathway of *y1* or *y9* (Li et al., 2007) and are also associated with β-carotene, zeaxanthin, and lutein in maize. Lycopene epsilon cyclase (*lcyE*) on chromosome 8 (Harjes et al., 2008) and β-carotene hydroxylase enzyme (*crtRB1*) also known as *BCH2* and *HYD3*, on chromosome 10 (Yan et al., 2010) have the most significant effect on provitamin A concentrations in the maize grains. As per Harjes et al. (2008), the gene *LcyE*, causes different variation in of concentration of carotenoids because of its four alleles affect ß−branches of the biosynthesis pathway of carotenoids. Three polymorphisms were identified in the gene *crtRB1*, which controlled the variations in carotenoids (Yan et al., 2010). There was a 5.2-fold increase in the carotenoid concentrations in the haplotypes, which possessed the favorable alleles of *crtRB1-50 TE* and *crtRB1-30 TE*. The gene *crtRB1* was identified to have a much greater effect on the concentrations of provitamin A than that of *LcyE* (Babu et al., 2013).

The gene *crtRB3*, which encodes the α-carotene hydroxylase enzyme (also called *BCH1*), is a major role player in the metabolic pathway of carotenoids in maize (Vallabhaneni and Wurtzel, 2009; Zhou et al., 2012). On chromosome 2, there is a QTL locus cluster that is associated with carotenoids (Table 7). The gene, *crtRB3*, was mapped on this QTL locus cluster. Eighteen polymorphic sites within *crtRB3* that are closely linked to the QTL cluster were found through candidate−gene association analysis using 126 diverse inbred lines of yellow maize. Significant effects on the level of α−carotene were noticed (from 8.7 to 34.8%) among the two SNPs, SNP1343 (in the 5’ untranslated region) and SNP2172 (in the second intron), with 1.7- to 3.7-fold differences. Recently, four QTLs namely, *qbc1-1, qbc5-1, qbc6-1*, and *qbc10-1* were mapped by Jittham et al. (2017) on three chromosomes (1, 5, and 6) of maize for β-carotene with 5.04 to 17.03 % phenotypic variation.

**TABLE 7:** QTLs/Genes associated with carotenoids and folate concentrations in rice, wheat, maize, and pearl millet.

Crop	Nutrient	QTL/gene	Chromosome	References
Rice	Folate	*qQTF-3-1, qQTF-3-2* and *qQTF-3-3*	3	Dong et al. (2013)
Wheat	*Carotenoid*	*Lute*	7	Ahmad et al. (2015)
Wheat	Provitamin A	*Psy-B1*	7	Pozniak et al. (2007)
		*Psy-A1*	7	He et al. (2008)
		*QYpc-1A, Qypc-1B, Qypc-4A*, and *Qypc-7A*	1A, 1B, 4A, and 7A	Zhang et al. (2009)
		*TaZds-A1*	2A	Dong et al. (2012)
		*AO1*, *AO2*, and *AO3*	2, 5, and 7	Colasuonno et al. (2017)
Maize	Folate	*q5-FTHFa* and *q5-FTHFb*	5	Guo et al. (2019)
Maize	Provitamin A	*lcyE*	8	Harjes et al. (2008)
		*crtRB1*	10	Yan et al. (2010)
		*crtRB3*	2	Vallabhaneni and Wurtzel (2009); Zhou et al. (2012)
		*Y1/PSY1*	6	Buckner et al. (1996)
		*PDS*	1	Li et al. (1996).
		*ZDS*		
		(*ZISO*)/*y9* locus	7	Chen et al. (2010); Matthews et al. (2003)
		*qbc1-1, qbc5-1, qbc6-1*, and *qbc10-1*	1, 5, 6, and 10	Jittham et al. (2017)
Maize	Lutein	*qtl^*l*^/umc1447–umc1692–umc2373*	5	Chander et al. (2008)
		*Qtl^*l*^ /phi091–atf2*	7	Chander et al. (2008)
		*qlut1-1* and *qlut6-1*	1 and 6	Jittham et al. (2017)
Maize	Zeaxanthin	*qtl^*z*^/phi30870–umc1553*	1	Chander et al. (2008)
		*qtl^*z*^/phi115–umc1735*	8	Chander et al. (2008)
		*ZEP1*		Vallabhaneni and Wurtzel (2009); Zhou et al. (2012)
		*qzea6-1*, *qzea8-1*, and *qzea10-1*	6, 8, and 10	Jittham et al. (2017)
		*PS1/LCYB*	5	Singh et al. (2003)
Sorghum	β-carotene	*Bc-1.1, Bc-2.1, Bc-2.2, Bc-2.3, Bc-10b.1*	1,2, 10b	Fernandez et al. (2008)

Zeaxanthin and lutein are the other major carotenoids that are found in maize. But, only a few QTLs/genes that are associated with zeaxanthin and lutein have been identified so far. The *Ps1* locus located on chromosome 5 was encoding *LCYB*. This locus is considered essential for the accumulation of zeaxanthin in maize (Singh et al., 2003). *ZEP1* is one of the major genes in the metabolic pathway of carotenoids in maize (Zhou et al., 2012). It controls the gene zeaxanthin epoxidase (Vallabhaneni and Wurtzel, 2009). Three QTLs, namely, *qzea6-1*, *qzea8-1*, and *qzea10-1*, explaining 12.5%, 6.7%, and 19.4% of phenotypic variation in zeaxanthin, are found on chromosomes 6, 8, and 10 of maize, respectively (Jittham et al., 2017). Jittham et al. (2017) identified two lutein QTLs that are mapped on chromosomes 1 and 6. They are designated as *qlut1-1* and *qlut6-1*, explaining 9.1 and 28.9 of phenotypic variation.

Folates are quantitative or polygenic traits typically controlled by several small effect QTLs. However, two major effect QTLs namely, *q5−F−THFa* and *q5−F−THFb*, explaining 26.7 and 14.9% of the folate variation were identified in maize (Guo et al., 2019) on chromosome 5 by whole−exome sequencing and F_3_ kernel−folate profiling. A unique correlation between the folate and the expression of the conserved genes of folate biosynthesis and metabolism was reported in the kernels of maize (Lian et al., 2015). Naqvi et al. (2009) and Liang et al. (2019) stated that the molecular understanding of the genetic networks of folates in grains is unclear even when successful increments have been made through transgenic experiments in maize.

### (d) Pearl Millet

The Pearl Millet inbred Germplasm Association Panel (PMiGAP) contains around 1000 accessions, cultivars, and landraces of pearl millet that have been collected from three major pearl millet growing continents (Sehgal et al., 2015). *Iniari* germplasms that are found on the landraces of West Africa have already been collected and stored along with other Indian landraces and cultivars by ICRISAT (Yadav et al., 1999). The USDA National Plant Germplasm System Pearl Millet Collection, presented at the Plant Genetic Resources Conservation Unit in Griffin, GA, United States, preserves about 1297 unique germplasm lines from around 31 countries. However, no efforts have been made to explore the genetic variation of carotenoids and folates in these germplasm collections. Thus, marker-assisted breeding to improve the carotenoids and folates suffer due to the lack of donors. There also appears to be a significant gap in the literature, as no genes/QTLs/markers have been discovered with an association with carotenoid and folate concentrations in pearl millet. The headway toward the genetic enhancement of pearl millet is still painstaking due to the lack of PCR-based co-dominant markers. It is hoped that the recently released reference genome of pearl millet will facilitate the discovery of markers/QTLs/genes associated with carotenoid and folate concentration. Also, researchers should consider synteny studies with other cereal crops to improve pearl millet nutritional breeding programs.

## Biofortification of Carotenoids and Folates in Cereal Grains

**FIGURE 4:**
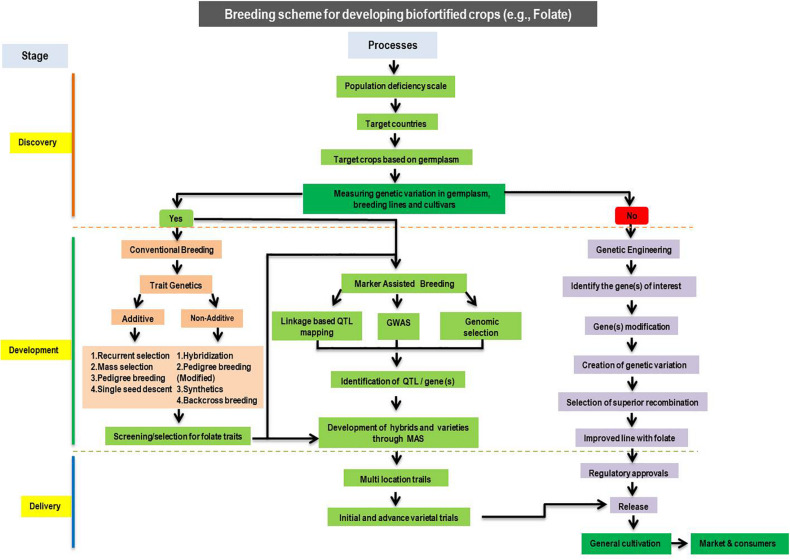
The proposed schema for developing biofortified cereal crops with enhanced nutrients (e.g., folates).

Biofortification is the process of increasing the natural content of bioavailable nutrients in plants. It is a successful and cost-effective method that associates nutritious agriculture with human health, can be efficient and more maintainable than the delivery of food supplements. Major tools in biofortification include conventional breeding, modern biotechnology, and agronomic practices (Figure 4). As mentioned above, carotenoids and folates are essentials for the human diet. Thus, biofortification of major cereal crops with carotenoids and folates may assist in easing micronutrient deficiencies in humans. Existing evidence recommends that genetic biofortification by breeding and modern biotechnology could be appropriate for increasing folates and pro-vitamin A carotenoids, and an agronomic strategy could be effective for Zn. Conventional breeding-based biofortification is the most successful approach to develop micronutrients rich crops, and several important food crops have been targeted for fortification by conventional breeding. So far, many more studies have been conducted to improve the provitamin A concentration and a few targeted at folate. Biofortification in maize has been attempted in many different ways. For instance, improvement of single or group of micronutrient (s) (single biofortification) and diverse micronutrients (double biofortification), including (i) the incorporation of favorable alleles of *crtRB1* and *lcyE* into popular elite genotype by MAB and transgenic approaches to increase the amount of provitamin A concentration and (ii) the development of genotypes with *crtRB1* and *lcyE* and *o2* alleles to increase the essential amino acids and provitamin A concentration by MAB (Hossain et al., 2019). In the recent decades, CIMMYT, Mexico, and IITA, Nigeria, developed and released many provitamin A varieties and hybrids (i.e., GV662A, GV664A, and GV665A, Ife maize hyb-3, and Ife maize hyb-4, Sammaz 38, Sammaz 39, and CSIR-CRI Honampa) in African countries (Dhliwayo et al., 2014; Simpungwe et al., 2017; Andersson et al., 2017). IARI released four provitamin A hybrids viz., HM4, HM8, and Vivek Hybrid-27 [which possessed provitamin A as high as 2170 μg/100 g (in freshly harvested grains) with a 8.5-fold maximum change] in India. The hybrid, “Pusa Vivek QPM 9 Improved,” which was developed through MAB, contains higher provitamin A (815 μg/100 g) even after storing for 2 months with higher levels of tryptophan, 0.74% and lysine, 2.67% (Muthusamy et al., 2014; Yadava et al., 2017). This hybrid was developed by the introgression of the *crtRB1* allele into a *o2-*based hybrid. In a similar manner, four popular QPM hybrids namely HQPM1, HQPM4, HQPM5, and HQPM7 were developed by pyramiding *crtRB1* and *lcyE* to improve the concentration of provitamin A (Hossain et al., 2019). Despite the success stories, conventional or marker-assisted breeding suffers due to the lack of genetic variation in micronutrient traits within the species or closely related species. In this context, transgenic technologies are an alternative to conventional breeding and useful to improve the genotypes by creating variations in targeted metabolic pathways. The concentration of provitamin A in rice was improved through transgenic methods. Over-expression of *PSY, CrtI* and *β-lcy* from daffodil, *Erwinia uredovora* and maize facilitated an increase in provitamin A concentration in rice lines (Ye et al., 2000; Beyer et al., 2002; Paine et al., 2005). In particular, *PSY* from maize increased provitamin A concentration up to 3700μg/100 g (Paine et al., 2005). Similarly, the contents of β-carotene increased to 1000 μg/100 g in the Hi-II maize line through the over-expression of *crtB* and *crtI* genes from *Erwinia herbicola* (Aluru et al., 2008). Likewise, five genes, namely, *psy1*, *crtI, lycb*, *bch*, and *crtW*, were used to develop transgenic maize genotypes that contained 6000 μg/100 g of β-carotene (Zhu et al., 2008; Naqvi et al., 2009). The over expression of *psy1* from maize and *crtI* or *CrtB* from the bacteria enhanced provitamin A to 496 μg/100 g and 321 μg/100 g of seed dry weight in wheat (Cong et al., 2009; Wang et al., 2014). Likewise, five genes, namely, *psy1*, *c rtI, lycb*, *bch*, and *crtW*, were used to develop transgenic maize genotypes that contained 6000 μg/100 g of β-carotene (Zhu et al., 2008; Naqvi et al., 2009). Despite the success of transgenic technologies, the main drawback to biofortified transgenic crops is their public acceptance and extensive regulatory processes required before they get clearance for cultivation and consumption by humans.

## Conclusion and Future Prospects

The growing world population requires many key nutrients and vitamins that can be delivered through staple foods. Advancing genomic tools can play an important role in accelerating genetic enhancement of these vitamins and minerals through biofortification in major cereal grains. Bioavailable vitamins or nutrients bred into varieties can be made available to resource-poor people generation after generation by their cultivation and regular consumption. The surplus production brings better livelihoods through marketing to other regions. Crop breeding requires substantial genetic variability and diagnostic markers to handle traits in segregating early generations. Nutrient-dense germplasm resources are essential to the breeding of adequate carotenoids and folates for fulfilling daily dietary requirements. National and international organizations have made excellent research progress in this direction to incorporate carotenoids into cereal crops. High throughput phenotyping tools (XRF, HPLC, and LC-MS/MS) are being developed and will be made accessible to partners at various organizations. These methods are cost effective for analyzing large sets of germplasm. The diagnostic markers play a key role in discarding low vitamin/nutrient materials. Integrating MAB creates the opportunity to introduce/track the QTL that are associated with nutritional quality into popular varieties. A survey of wild and cultivated accessions demonstrated noticeable variations in the carotenoid and folate concentration and the possibility to identify novel sources for alleles to be used to broaden the present gene pool. So far, substantial genetic variation has been exhibited only in the genetic resources of maize for provitamin A. Other major cereals, like rice, wheat, and pearl millet commercial or elite lines, lack sufficient concentration of provitamin A to achieve global target levels. Almost no folate research has been done in major cereal crops. Biofortification based breeding has been demonstrated as a successful of enhance the micronutrients in cereals. However, new breeding designs, such as MAGIC populations and GS, also need to be explored on parallel to maximize the genetic understanding and identification of QTLs and genes for complex traits such as carotenoids and folates. Hence, greater prospects await with the use of these technologies in nutrition breeding. On the other hand, where inadequate genetic variability exists within the cultivated germplasms and primary gene pools, then the transgenic technology may be an option for enhancing carotenoids and folates in cereals but has limited scope for acceptance in most of the developing countries. Genetic gain for yield alone may not be appropriate to feed the growing population, but concurrently achieving nutrition traits genetic gains is a sustainable approach. Government programs are required to create public awareness for the adoption of biofortified varieties by farmers through increased consumer acceptance. Moreover, research coordination is required between agriculture and nutritional experts for strengthening the target level of carotenoids and folates, their retention after cooking, storage, processing, and consumption of prospective concentrations in the target population. Therefore, with the available genetic resources and genomic tools, breeding investment should be made and optimized for increasing vitamins and nutrients in staple food crops besides increasing sustainable yields.

## Author Contributions

MG and KA conceptualized the manuscript. KA, MG, AK, and VS wrote the manuscript. MG and TW edited and updated the manuscript.

## Conflict of Interest

The authors declare that the research was conducted in the absence of any commercial or financial relationships that could be construed as a potential conflict of interest.
